# Microbial Interactions between Marine Microalgae and Fungi: From Chemical Ecology to Biotechnological Possible Applications

**DOI:** 10.3390/md21050310

**Published:** 2023-05-19

**Authors:** Chiara Lauritano, Christian Galasso

**Affiliations:** 1 Department of Ecosustainable Marine Biotechnology, Stazione Zoologica Anton Dohrn, Via Acton n. 55, 80133 Naples, Italy; 2 Department of Ecosustainable Marine Biotechnology, Calabria Marine Centre, Stazione Zoologica Anton Dohrn, C. da Torre Spaccata, 87071 Amendolara, Italy

**Keywords:** microalgae, fungi, microbial interactions, drug discovery, defensive compounds, symbiosis, chemical communication, chemical ecology, green procedures, bio-flocculation, bioremediation

## Abstract

Chemical interactions have been shown to regulate several marine life processes, including selection of food sources, defense, behavior, predation, and mate recognition. These chemical communication signals have effects not only at the individual scale, but also at population and community levels. This review focuses on chemical interactions between marine fungi and microalgae, summarizing studies on compounds synthetized when they are cultured together. In the current study, we also highlight possible biotechnological outcomes of the synthetized metabolites, mainly for human health applications. In addition, we discuss applications for bio-flocculation and bioremediation. Finally, we point out the necessity of further investigating microalgae-fungi chemical interactions because it is a field still less explored compared to microalga–bacteria communication and, considering the promising results obtained until now, it is worthy of further research for scientific advancement in both ecology and biotechnology fields.

## 1. Introduction

Marine chemical ecology is the integrative science which studies the ecology and evolution of marine populations, marine communities, and functioning of marine ecosystems [1,2,3]. In particular, it focuses on chemical signals which represent the life language in the sea. In the last 20–30 years, several papers have shown how metabolites produced by marine organisms influence feeding choices, organism associations, and mating, by impacting the transfer of energy and nutrients within and among ecosystems [3].

Chemical signals have been shown to regulate/influence inter- and intra-specific interactions not only at individual scale, but also at population and community levels [4]. In particular, the marine environments are characterized by a huge chemical diversity, which includes primary and secondary metabolites, as well as potent toxins inducing damage to humans till their death [5,6,7]. According to the MarinLit database (https://marinlit.rsc.org/; assessed on 8 March 2023), there are currently 39,553 articles on MNPs and 39,897 known compounds. However, the possible functions and ecological roles of each compound are still not known.

Many MNPs have been shown to influence a plethora of key life processes, such as the identification and selection of food sources, chemical defense (e.g., antipredator, antibacterial and allelopathic functions), behavior, localization and capture of possible prey, and mate recognition [1,2,3,4]. The activities/roles of MNPs directly and indirectly influence interactions between individuals in the sea, whether belonging to the same species or not, as well as marine biodiversity and ecosystem functioning. MNPs do not have to be considered as one compound–one activity, as they may exert multiple and simultaneous functions, acting with different modes of action [4]. For example, diatom polyunsaturated aldehydes (PUAs) are known to have antipredator activity against copepods and other invertebrates [8,9], allelopathic effects on other microalgae [10], and they have been shown to alter bacterial growth and species composition [11]. Altogether, these effects may modify species abundance and distribution at sea, eventually shaping the marine food web.

In addition to their ecological roles, PUAs have been shown to exert antiproliferative activity against colon carcinoma cell lines, highlighting how MNPs can also be biotechnologically exploited [12,13,14]. PUAs have also shown the capability of reducing abalone infestations by shell-boring polychaete worms [15]. Not only may one molecule have different activities on various marine organisms, but each organism is also continuously exposed to a bouquet of compounds produced by different organisms. This chemical communication is at the basis of the marine life functioning. Another example regards shape modifications. For instance, the bloom-forming phytoplanktonic species *Phaeocystis globosa* is able to sense surroundings [16]. In fact, when there are ciliates that feed on small food, *P. globosa* changes shape and grows as a colony, while when in the presence of copepods that feed on larger food, it grows as single cells.

Between marine microorganisms, the most studied interactions are those between microalgae and bacteria [17,18]. However, recently, researchers have also focused on the microalgae–fungi symbiosis. The aim of this review is to summarize known microbial interactions between marine microalgae and fungi, reporting key communication chemicals involved as well as their possible biotechnological applications.

## 2. Microalgae and Fungi Interactions

Marine microalgae and fungi play key roles in microbial food webs and biogeochemical cycles. They cooperate using several interspecific mechanisms to exchange nutrients and information through signaling molecules. Symbiosis among two group of microorganisms living in the same habitat allows to regulate basic mechanisms, such as predation, nutrient search, and proliferation.

In the last decades, the so-called mycophycobioses raised strong interest, focusing research attention on stable symbiotic relationships between fungi and microalgae, probably considered primitive lichenizations. Marine mycoplankton can establish a saprophytic, pathogenic, defense, competition, and parasitic relationship with microbial communities. However, few information and scientific data are available on the chemical ecology regulating microalgae–fungi communication, even if a considerable number of studies have been published in recent years mainly on saprophytic and parasitic microalgae-fungi relationship. However, the different phases of fungi-microalgae interaction and the molecules involved remain unclear and poorly studied, although several types of interspecific relationships have been observed and described so far.

Microalgae have been found widely distributed in multiple habitats, including extreme environments. They account for the 40% of annual primary productivity in the ocean carbon cycle [19] and represent up to 25% of global carbon-fixation [20]. Similarly, marine fungi have been found in almost all marine ecosystems explored, from superficial waters to deep sediments. Fungi were found in association with phytoplankton population, contributing to the exchange of inorganic and organic matter and to the biological carbon pump. They can use monomers from the degradation of microalgal polymeric substrates to increase their growth rate [21].

### 2.1. Microalgae–Fungi Saprophytic Relationship

Fungi are known to develop saprophytic interaction with eucaryotic living organisms. They can obtain carbon from dying cells or from the decomposition of organic matter. Among diatom-derived substrates utilised by marine saprophytic fungi, polysaccharides constitute the best example and the most investigate group of biopolymers. For instance, *Phaeodactylum tricornutum* can produce and release in the medium chrysolaminarin, a glucan with high chemical similarity to laminarin (Table 1).

Samples coming from Tara Ocean Expeditions [27] were used to isolate saprotrophic mycoplankton selected for their ability to utilise laminarin as a substrate under in vitro controlled conditions. *Cladosporium* spp. can synthetize the carbohydrate-active enzyme glucan 1,3-*β*-glucosidase, providing evidence that species of this hyphomycete genus utilize microalgal derived polysaccharides as well as information on the role of mycoplankton in the carbon transfer to the higher tropic levels [22].

Among microalgae, dinoflagellates are a group of phytoplanktonic microorganisms that can have a deleterious impact on marine ecosystems with negative effects on the growth and diversity of other marine species. Dinoflagellates are responsible for harmful algal blooms (HABs) where high level of toxins can be produced, e.g., okadaic acid [23]. To study the dinoflagellates-fungi interactions under controlled conditions, Berry and collaborators set up a miniaturized liquid-solid environment, where fungal strains grew on f/2 GA solid medium and dinoflagellates in f/2 liquid medium. In particular, they studied the microalga *Prorocentrum lima* PL4V strain and the fungus *Aspergillus pseudoglaucus* MMS1589 strain, and evaluated, through a metabolomic approach with high-performance liquid chromatography coupled to high-resolution mass spectrometry (LC-HRMS), the biosynthesis of compounds produced as single culture, in the co-culture, as axenic or not, in agar or liquid media. They found 51 features over-produced during the co-culture, of which 14 were also detected in the microalgal monoculture, six in the fungal monoculture, while 34 were only observed in the co-culture. Of these compounds, 12 were found in agar medium and 42 in liquid medium. Interestingly, greater differences were found for *P. lima* metabolomes under axenic and not culturing conditions, compared to fungal one. Hence, the chemical induction was stronger in the presence of bacteria. Some of the compounds were found only when antibiotics were added (used to make the algal culture axenic) suggesting the fungal–algal-interaction. Okadaic acid (OA) and its derivative dinophysistoxin 1 (DTX-1) (Table 1) were produced by the dinoflagellate during co-culture conditions (with or without antibiotics), together with two toxins usually involved in the *P. lima* toxicity, in response to the presence of *A*. *pseudoglaucus*. The production of such toxins can be read as a defense mechanism activation of the dinoflagellate to regulate and control the fungal growth [28]. In addition, *A*. *pseudoglaucus* probably responded to the stressful condition by synthetizing 5*S*,8*R*-Dihydroxy-9*Z*,12*Z*-octadecadienoic acid (Table 1), which was already described as produced by *Aspergillus* spp. The study also described a physical interaction between the two species by using microscope analysis, highlighting an unusual flagellum–mycelium contact.

### 2.2. Microalgae–Fungi Parasitic Relationship

Parasitic interaction is established when only an organism benefits from the symbiosis to the detriment of the other/s. Parasites cover important ecological roles in many marine ecosystems, since they can modulate microorganisms’ communities, food web structure, and nutrient availability [29]. One of the most abundant groups of parasites in freshwater and marine environments is represented by chytrids (Chytridiomycota), aerobic zoosporic fungi that act as saprotrophs and pathogens for phyto- and zooplankton, but also for macroorganisms, e.g., plants and invertebrate animals [30]. The zoosporic stage of their life cycle is constituted by free swimming zoospores constantly looking for new host cells. Sporangia are the mature stage that use host nutrients to develop and secrete new zoospores [31]. The parasitic interaction between chytrids and diatoms has been investigated only in the recent years. A study of Scholz and collaborators stabilized co-culture of chytrids and diatoms under controlled conditions to study the influence of some abiotic factors on the first parasite–host interaction and the variation of signal molecules involved in the diatom defence strategy [24]. Chytrids can recognize host cells by using chemotactic signals, driving motility toward available nutrients and host cells. In the study by Scholz et al., chemiotaxis was evaluated for four chytrids types: *Chytridium* sp., *Rhizophydium* type I, *Rhizophydium* type IIa, and *Rhizophydium* type IIb. Chemiotactic stimuli were constituted by standard macromolecules, i.e., carbohydrates, amino acids, fatty acids, quaternary ammonium compounds, and polyol glycerol, and total extracts of diatom biomasses (*Navicula* sp., *Nitzschia* sp., *Rhizosolenia* sp. and *Chaetoceros* sp.). All the chemotactic signals investigated on the zoospores gave a positive response, varying in the intensity of attraction. The highest chemiotactic response was obtained with diatom total extracts, especially when microalgal culture was characterised by light stress conditions. Moreover, a comparative analysis between chemical composition of diatom resistant strains and susceptible ones revealed that aldehydes and PUFAs (Table 1) were highly concentrated in the biomass and medium of resistant diatom taxa, giving an insight in their involvement in the diatom defence strategy against parasites [24].

Oomycetes represent a group of parasitic eukaryotic organisms with unique molecular pathways involved in host infection. They are classified as fungi due to their filamentous growth, even if recent molecular taxonomic characterization and biochemical studies also suggest high affinity with brown algae kingdom [32]. The parasitic infection of the oomycetes *Langenisma coscinosdisci* on marine diatoms species of the genus *Coscinodiscus* is well documented. These diatoms are known to create abundant seasonal blooms along cold coastal waters of the Northern Europe, and oomycetes infections represent a biotic factor that can strongly influence and regulate proliferation and ecology interactions of these phytoplanktonic microorganisms [33,34]. Interactions between *L. coscinodisci* and diatoms of the genus *Coscinodiscus* already represent a strengthened experimental model for the study of phytoplankton-parasites symbiosis. A recent study identified two indole alkaloids (4-carboxy-2,3,4,9-tetrahydro-1H-*β*-carboline, 4-CTC and *β*-carboline; Table 1) synthetized by the diatom *Coscinodiscus granii* when infected by *L. coscinodisci*. These compounds are responsible of the reduction of microalgal growth rate and induction of plasmolytic response, through vacuolization and cytoplasmatic detachment in diatom cells. 4-CTC and *β*-carboline are synthetized when tryptophane pathway is triggered. These two metabolites were found to be up-regulated in infected diatom cells [26] and they induced in vitro reduction of *C. granii* proliferation when added to culture medium, promoting *L. coscinodisci* infection. In addition, the results demonstrated that *L. coscinodisci* not only infect diatoms, but also modulate the cell metabolome during its life cycle. All these findings were in accordance with previous results, where researchers studied plant–oomycete interaction, showing the stimulation of the host immune system through *β*-1,3-glucans and eicosapolyenoic acid activity [35].

### 2.3. Microalgae–Fungi Microbial Competition

Microorganisms constantly activate intra- and inter-specific mechanisms in the competition for spaces, nutrients, and other chemo-physical factors in their surroundings. These interactions activate biosynthesis of chemical signals that can deeply shape microbial communities. A recent study contributed to supply additional evidence for the ecological model in which fungi have a role in the regulation of phytoplanktonic community in coastal upwelling marine ecosystems [36]. Through a long-term sampling along the coast of Chile, researchers identified attached Chytridiomycota on strains belonging to the diatom microalgal genera *Skeletonema* and *Thalassiosira*, by using electron microscopy. These groups of microalgae generally follow a seasonal variability, with peaks of abundance during austral spring and a reduction in summer and winter [37]. Similar seasonal variability was observed for chytrid abundance, with the highest concentration during austral spring and summer. Interestingly, attached sporangia reached maximum concentration during austral spring, in conjunction with *Skeletonema* and *Thalassiosira* bloom, while detached chytrid sporangia followed the opposite dynamic, with the highest concentration during late spring and summer. These data strongly suggest that these two diatom genera are suitable target hosts for Chytridiomycota free living zoospores, showing how fungi can deeply shape the microalgal community in a marine environment [36].

*Aspergillus* species are ubiquitous in both terrestrial and marine environments and have been demonstrated as pathogenic to a wide range of organisms [38]. Among them, *Aspergillus sydowii* inhabits terrestrial environments, but a sporogenic stage of its life cycle can occur marine ecosystems, where it has been described as a causative agent of aspergillosis of corals belonging to the genus *Gorgonia*. *A. sydowii* can create necrotic areas in coral tissues, re-shaping the endosymbiotic community. *Symbiodinium* dinoflagellate endosymbionts were negatively regulated in terms of abundance in the presence of secondary metabolites synthetized by *A*. *sydowii*. The most active compounds were elucidated (Table 1) as sydowinin A and B, sydowinol, and sydowic acid [25].

An interesting ecological triple association is represented by the fungus *Aspergillus nidulans*, the unicellular green alga *Chlamydomonas reinhardtii*, and the bacterium *Streptomyces iranensis*. Close interaction between microalga and bacterium triggers in the latter the biosynthesis of the algicidal molecule azalomycin F. In a co-cultivation system, the microalga *C*. *reinhardtii* established a cell–cell interaction with the fungus *A*. *nidulans*, producing microalgal cells completely surrounded by the mycelium. This structure created a protection against the toxic action of the azalomycin F [39]. These findings provided insights on the evolutionary selection of this microalga–fungus symbiosis, appearing as a strategy to counteract toxic chemical stimuli in the marine environment.

## 3. Possible Biotechnological Applications

### 3.1. Compounds for Human Health Applications

The large diversity of biotic stimuli interconnecting between marine microalgae and fungi is regulated by the trophic relationship and is carried out by diffusible signaling molecules. Microalgae-fungi symbiosis has receiving increasing attention for the ecological role of these microorganisms in marine environment and for potential use of metabolites and processes activated during co-cultivation in industrial applications. Marine microalgae and fungi often inhabit the same ecological niche, living in both the water column and benthic substrates. Such close growth has developed a plethora of physical and chemical interactions poorly explored from ecological and biotechnological point of view.

**Figure 1:**
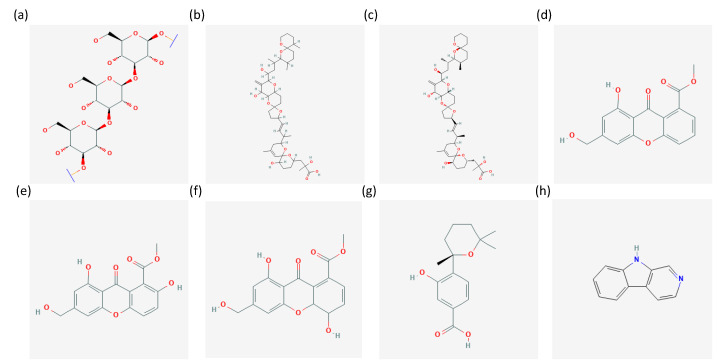
Chemical structures of (**a**) Chrysolaminarin [53], (**b**) okadaic acid [54], (**c**) dinophysistoxin 1 [55], (**d**) sydowinin A [56], (**e**) sydowinin b [57], (**f**) sydowinol [58], (**g**) sydowic acid [59], (**h**) *β*-carboline [60] from the PubChem database.

Chrysolaminarin (Figure 1) is a storage polysaccaride, consisting of a *β*-1,3-linked backbone with infrequent *β*-1,6-linked branches, typically found in microalgae [40]. It has been reported, in fact, in the diatoms *Phaeodactylum tricornutum* and *Odontella aurita* [40], in the Chrysophytes *Poterioochromonas malhamensis*, *Isochrysis zhangjiangensis* and *Isochrysis galbana* [41,42,43], and in the Eustigmatophyte *Nannochloropsis gaditana* [44]. It is a hydrophilic compound, stored in the vacuoles. It has been reported to also reach more than 40% of algal biomass when cultured in specific conditions, such as nutrient deprivation [43], suggesting a preference for stressful culturing conditions in order to easily accumulate, extract, and purify it [40,42]. Regarding chrysolaminarin bioactivity, it has been reported to exert antioxidant, immunomodulatory, anticancer and regeneration-promoting effects [41,42,45,46]. In particular, Sadovskaya et al. [41] reported that chrysolaminarin inhibited in vitro the proliferation of human leukemic monocyte lymphoma cells (U937), while other data described this compound as able to promote fin regeneration and improve in vivo antioxidant activity on zebrafish [42]. Antioxidant activity was also reported in vitro by other studies which described chrysolaminarin able to scavenge 1,1-diphenyl-2-picrylhydrazyl (DPPH) radical [45] and by ORAC and FRAP assays [46]. Finally, intraperitoneal injections of juvenile soles with the chrysolaminarin-enriched extract were used to evaluate its possible immunomodulatory activity. Results showed the activation of a series of genes involved in the inflammatory response. For this reason, the authors proposed this chrysolaminarin-enriched extract as immunostimulating feed in aquaculture [46]. Thanks to these properties, it is considered a good candidate for food and pharmaceutical applications [43].

5*S*,8*R*-Dihydroxy-9*Z*,12*Z*-octadecadienoic acid was reported as produced by the fungus *Aspergillus pseudoglaucus* MMS1589 exposed to *P. lima*. In the literature, octadecadienoic acids have been reported to exert anticancer activity [47], but to our knowledge, the bioactivity of 5*S*,8*R*-Dihydroxy-9*Z*,12*Z*-octadecadienoic acid has not yet been described. The heterologous expression in *Escherichia coli* has been optimized for this compound [48] and recombinant cells exhibited a conversion yield of 99% (converting linoleic acid into the desired product) and a productivity of 2.5 g L^−1^ h^−1^.

Recently, Berry et al. performed an experiment involving the co-culturing of microalgae and fungi, as well as their single cultures, but also evaluated the effects of the algal associated bacteria (by using axenic and not axenic algal cultures). They found between the compounds produced by the microalga *Prorocentrum lima*, induced by the co-culture (with or without antibiotics), okadaic acid (OA) and its derivative dinophysistoxin 1 (DTX-1) (Figure 1) [23]. As reported by Camacho et al. [49], marine toxins, including those produced by dinoflagellates, may have biotechnological applications, e.g., as topical anesthetics. Several studies are focusing the attention on their possible applications, especially in the pharmaceutical field [50]. OA, in particular, has been found to inhibit the protein phosphatase 2A and was suggested for possible breast anticancer applications [51]. OA has been also studied as a lead compound to develop possible drugs for the treatment of schizophrenia and other neurodegenerative diseases [52].

Regarding aldehydes and PUFAs, both have been previously reported to exert anticancer properties. Polyunsaturated aldehydes (PUAs) are able to induce programmed cell death in marine organisms, such as sea urchins, and a similar effect was also found on human cells [61]. For example, Miralto et al., in 1999, showed that PUAs were able to suppress the growth of human colon carcinoma Caco-2 cells and activate apoptosis [12]. Similarly, Sansone et al. showed that PUAs were able to induce cell death when tested on lung A549 and colon COLO 205 adenocarcinoma cell line [62]. Regarding PUFAs, eicosapentaenoic acid (EPA) and docosahexaenoic acid (DHA) bioactivities have often been studied [63]. DHA has been reported to have anticancer properties against breast (MDA-MB-231, MCF-7), pancreatic (MiaPaca-2), and colorectal (CaCo-2, SW-620) cancer cell lines, whereas EPA against lung human A549 and human esophageal cancer cell lines. EPA, in addition, has been also reported for its antibacterial properties against *Bacillus cereus* and *Staphylococcus aureus*.

Sydowinin A and B, sydowinol, and sydowic acid (Figure 1) have been tested and found to exert cytotoxicity on human intestinal (HT-29) and neuroblastoma (SH-SY5Y) cell lines [64]. Sydowinol was the most active with IC_50_ of 2.50 µM and 5.14 µM, respectively. The activity of sydowic acid was considered not significant within the tested concentration range. Similarly, Yao et al. [65] have shown activity against human histiocytic lymphoma U937 cell line for Sydowinin A and B (IC_50_ of 75.6 and 127 µM, respectively). Sydowinin B have also shown antibacterial properties (against *Vibro rotiferianus*), while both sydowinin A and B showed immunosuppressive properties, finding possible applications in the pharmaceutical sector [66,67].

Finally, other metabolites found involved in microalgae-fungi interactions were 4-carboxy-2,3,4,9-tetrahydro-1H-*β*-carboline (4-CTC) and *β*-carboline (Figure 1). *β*-carbolines are a group of alkaloids reported to have anticancer properties. They have been shown to act by activating various mechanisms of action, including the inhibition of DNA topoisomerases I and II, intercalation to DNA, or affecting various cancer signaling pathways [68,69,70]. Considering its high risk of cytotoxicity to normal cells, various analogues have been studied, such as the tetrahydro-*β*-carboline, which have shown lower cytotoxicity [71,72,73]. In addition to anticancer properties, some *β*-carbolines have been also reported to have antioxidant, neuroprotective, and anti-inflammatory effects [74].

### 3.2. Bio-Flocculation

Microalgae are known to produce a huge portfolio of bioactive compounds with applications in different industrial sectors [75,76,77,78]. However, productivity still imposes high costs for specific operation procedures, such as harvesting, nutrient supply, and compound extraction. Harvesting, in particular, still requires high-energy input and is a limiting factor for possible eco-sustainable microalgal uses. The in vitro interaction between microalgae and fungi can be also exploited to simplify and reduce the cost of microalgae harvesting. In recent years, a phenomenon called “bio-flocculation” has attracted attention for the industrial exploitation of such small and low density microalgal cells. Bio-flocculation is a strategy that uses biomolecules or microorganisms for the creation of cell aggregates (flocs) in a liquid medium. Flocs drastically reduce time and costs for microalgal biomass harvesting [79]. For instance, *Nannochloropsis* species are reported to have good biotechnological potential for lipid production (for biofuel) and for high-values compound biosynthesis (e.g., polyunsaturated fatty acids, PUFAs). A huge obstacle to the industrial use of these microalgal species is represented by high costs for harvesting cells, due to the small cell size (from 2 to 20 µm) growing in low density (lower than 5 g × L^−1^) [80]. Marine fungi can be used, in co-culture condition, for the bio-flocculation of microalgae [81], avoiding the use of chemical flocculants or high-energy harvesting techniques. *Nannochloropsis oceanica* CCMP1779 can create flocs when grown with the oleaginous fungus *Mortierella elongata*. This co-culture condition, after six days, created green clusters of microalgal cells on the mycelium of the fungus [80]. The *N*. *oceanica* floc growth showed specificity with *M*. *elongata*, since it was not observed with other fungal strains tested. In addition, this microalga–fungus interaction induced an increment of triacylglycerol, PUFAs and total fatty acids production. These two species naturally produce oil and high values compounds, but in a symbiotic relationship the biosynthesis of this biotechnological interesting compounds get increased. Symbiosis between *N*. *oceanica* and *M*. *elongata* is not characterised only by a physical interaction, but also by a nutrient exchange. Isotope trace experiments have demonstrated a flow of carbon in the microalga-fungus direction and a flow of nitrogen in the fungus-microalga direction [80]. After a long term co-cultivation, this symbiosis became highly stable, since the metabolism of the two partners was observed to be physiological active and *N*. *oceanica* cells did not only adhere to mycelium, but seemed to be internalised within *M*. *elongate* hyphae, remaining viable [80].

Wrede et al. [82] also highlighted how studies on microalgae–fungi co-cultivation are attracting attention for the increased bio-flocculation of the microalgal strains with low energy inputs. In addition, bio-flocculation by co-culturing does not require chemicals and is therefore considered a green approach. Wrede et al. tested the flocculation efficiency of the fungus *A*. *fumigatus* on 11 microalgae, including species collected in marine and freshwater habitats, heterotrophic and photoautotrophic, as well as species of different cellular dimensions and motility. In particular, they were the freshwater species *Chlorella vulgaris*, *Chlamydomonas reinhardtii*, *Pseudokirchneriella subcapitata*, and *Scenedesmus quadricauda*, and the marine species *Thraustochytrid* sp., *Dunaliella tertiolecta*, *Dunaliella salina*, *Nannochloropsis oculata*, *Nannochloris oculata*, *Tetraselmis chuii*, and *Pyrocystis lunula*. Results showed that co-cultivation allowed to get synergistic effects on biomass production. In terms of applications, they found an increase in lipid yield and wastewater bioremediation efficiency. Microalgal lipids have found different biotechnological applications, including nutraceutical components, cosmetic ingredients, and for biodiesel production [75].

### 3.3. Fungi–Microalgae Consortia for Wastewater Treatment

Microalgae are well studied organisms for the possible removal of toxic contaminants from wastewater, since current chemical decontamination methods are no longer considered eco-sustainable approaches both from the ecological and economical points of view. In the last decade, consortia of microalgae and fungi have come to represent a new biological model for the exploitation of marine microorganisms for environmental application. In fact, the co-cultivation of fungi and microalgae bypasses some obstacles encountered with the use of a single strain decontamination system. Properties, such as cell size and harvesting, surface charge, growth rate, and motility, can be ameliorated under controlled co-culture conditions, exploiting a synergistic effect exerted by the two strains, thus increasing the decontamination rate and biotechnological success [83].

As reported above, the co-cultivation of microalgae and filamentous fungi facilitate the harvesting methods, since the fungi-based flocculation allows to increase the size of particles to be collected (0.5–2 cm respect to 0.001–0.01 cm of only microalgal cells), reducing costs (no chemical flocculants are needed) and time. A possible mechanism of association is based on negative microalgal external wall and positive charged fungi that establish electrostatic interactions. Another interaction is regulated by fungal hyphae that are rich in exopolysaccharides (EPSs), creating a highly adhesive substrate for microalgal cells. This cell–cell interaction is similar to the microalgae-bacteria consortia, where EPSs constitute the anchoring biopolymers for the formation of flocs [84]. Fungal–microalgal pellet formation is a process that can be regulated by culture conditions, including pH, nutrients, agitation, and aeration, thus constituting a composite industrial procedure [85]. Porosity is another factor that strongly influence flocs industrial application. While microalgae-bacteria consortia showed low porosity, reducing the interaction with nutrients and oxygen, fungi confer high porosity to microalgae-fungi flocs, promoting their potential use in the wastewater treatments at industrial level [84,86]. In addition, a co-cultivation system enlarges the number and the chemical diversity of extracellular macromolecules, enzymes, and biopolymers that can interact, adsorb, and degrade pollutants in wastewater [82].

Wastewater contains many toxic organic compounds that can be used from microorganisms for their metabolic processes as carbon source. Different combinations of microalgal and fungal species have been studied in co-cultivation systems, as well as each one alone, to understand if the combination was able to increase the COD removal rate (Chemical Oxygen Demand is an index for the quantification of organic pollutants in waste matrices). A mixotrophic condition favourites both the microalgal and fungal growth, through exchange of gas and carbon sources [21,82]. Microalgae use carbon dioxide for their metabolism, releasing oxygen and organic compounds used by fungi. This flux of molecules augments and facilitates the utilization of carbon from wastewater.

In addition, fungi-microalgae consortia can be used to remove nutrients from wastewater, producing oil-rich biomass for biofuel application [87]. Yang et al. [87] described the improvement in removal of COD from molasses wastewater when a co-culture system was used. In fact, only microalgae (*Chlorella vulgaris*) removed 26%, only fungi (*Aspergillus* sp.) 59%, while the co-culture reached 71% removal. Similar results were obtained in the removal of total phosphorus (TP), which increased up to 88%. Total nitrogen (TN) was removed from wastewater in low concentrations from only microalgae (44%) and fungi (18%), while the co-cultivation strategy brought 67% removal. Finally, the food cycle established between these two microbial strains led to a higher level (35%) of lipid content (with respect to monoculture system) making produced biomass suitable for biofuel production [87].

## 4. Discussion

Marine organisms are known to communicate under water by using chemical signals. They are in fact known to produce a plethora of compounds with great diversity in terms of activities, chemical structures and quantities [75,88,89,90]. Differences also depend on intra- and inter-species variability and induction by biotic and abiotic stimuli [91,92]. This communication has not only been reported for microorganisms, but also for their predators and higher trophic levels. For example, the copepod *Acartia tonsa* is known to produce compounds, polar lipids, named copepodamides, which induce paralytic shellfish toxin production in the marine dinoflagellate *Alexandrium minutum* [93].

**Figure 2:**
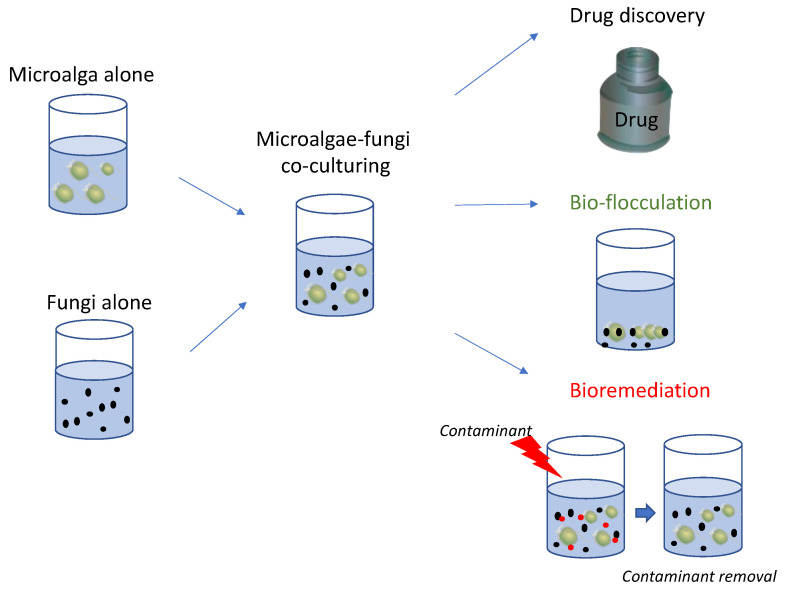
The figure reports a schematic representation of marine microalgae-fungi co-culturing and possible applications in various industrial fields as reported in literature, including the identification of chemical mediators for drug discovery, improvement of microalgal harvesting procedures (bio-flocculation) and removal of contaminants (bioremediation).

Microalgae and bacteria co-culturing have shown different possible outcomes, ranging from the production of bioactive molecules [94] to improvement in harvesting methods (i.e., bio-flocculation [95]) and bioremediation [96]. Each one represents advancements in ecological and biotechnological knowledge. The current review reports the molecules produced by co-culturing marine microalgae and fungi, such as chrysolaminarin, okadaic acid, dinophysis toxin 1, PUFAs, sydowinin A and B, sydowinol, sydowic acid, and *β*-carboline, which have been shown to exert immunosuppressive, anti-inflammatory, antioxidant, and neuroprotective properties. In addition, many of these compounds have been reported to have anticancer activities against various human cell lines, including breast, pancreatic, neuroblastoma, and colorectal cells. These bioactivities testify that these compounds can be of interest for the pharmaceutical, nutraceutical, and cosmeceutical sectors (Figure 2).

Various studies have reported that their co-culturing also provided good results in terms of bioremediation, such as for molasses wastewater treatment, and total nitrogen and phosphorus removal (Figure 2). Comparisons with microalgae and fungi alone highlighted the improvements obtained by culturing them together for contaminant removal.

Finally, their co-culturing also allowed to obtain excellent results in reducing the time and costs for microalgal harvesting, one of the most expensive and time-consuming steps of their culturing. These positive results can be of interest at industrial scale as well, by reducing the use of chemicals for inducing flocculation, using more green approaches and saving energy by avoiding high energy consuming steps.

Hence, the co-cultivation of microalgae and fungi can be very useful for an eco-friendly exploitation of the marine resources. Altogether, these data suggest the necessity of further exploring microalgae and fungi interaction, still less explored compared to other marine organisms, in order to speed up the finding of green solutions to various health and societal needs.

## Figures and Tables

**Table 1:** This table summarizes compounds biosynthesized by microalgae or fungi when they are co-cultivated.

Compound	Producer	Interaction with	Reference
Chrysolaminarin	Diatom *Phaeodactylum tricornutum*	Fungus *Cladosporium* spp.	[22]
Okadaic acid and Dinophysistoxin 1	Dinoflagellate *Prorocentrum lima* PL4V	Fungus *Aspergillus pseudoglaucus* MMS1589	[23]
5*S*,8*R*-Dihydroxy-9*Z*,12*Z*-octadecadienoic acid	Fungus *Aspergillus pseudoglaucus* MMS1589	Dinoflagellate *Prorocentrum lima* PL4V	[23]
Aldehydes and PUFAs	Diatoms	Chytrids	[24]
Sydowinin A and B, sydowinol and sydowic acid	Fungus *Aspergillus sydowii*	Dinoflagellates *Symbiodinium* spp.	[25]
4-carboxy-2,3,4,9-tetrahydro-1H-*β*-carboline (4-CTC) and *β*-carboline	Diatom *Coscinodiscus granii*	Fungus *Langenisma coscinosdisci*	[26]

## Data Availability

Not applicable.
